# Competition influences tree growth, but not mortality, across environmental gradients in Amazonia and tropical Africa

**DOI:** 10.1002/ecy.3052

**Published:** 2020-05-05

**Authors:** Danaë M. A. Rozendaal, Oliver L. Phillips, Simon L. Lewis, Kofi Affum‐Baffoe, Esteban Alvarez-Davila, Ana Andrade, Luiz E. O. C. Aragão, Alejandro Araujo‐Murakami, Timothy R. Baker, Olaf Bánki, Roel J. W. Brienen, José Luis C. Camargo, James A. Comiskey, Marie Noël Djuikouo Kamdem, Sophie Fauset, Ted R. Feldpausch, Timothy J. Killeen, William F. Laurance, Susan G. W. Laurance, Thomas Lovejoy, Yadvinder Malhi, Beatriz S. Marimon, Ben‐Hur Marimon Junior, Andrew R. Marshall, David A. Neill, Percy Núñez Vargas, Nigel C. A. Pitman, Lourens Poorter, Jan Reitsma, Marcos Silveira, Bonaventure Sonké, Terry Sunderland, Hermann Taedoumg, Hans ter Steege, John W. Terborgh, Ricardo K. Umetsu, Geertje M.F. van der Heijden, Emilio Vilanova, Vincent Vos, Lee J. T. White, Simon Willcock, Lise Zemagho, Mark C. Vanderwel

**Affiliations:** ^1^ Department of Biology University of Regina 3737 Wascana Parkway Regina S4S 0A2 Saskatchewan Canada; ^2^ Laboratory of Geo‐Information Science and Remote Sensing Wageningen University P.O. Box 47 6700 AA Wageningen The Netherlands; ^3^ Forest Ecology and Forest Management Group Wageningen University P.O. Box 47 6700 AA Wageningen The Netherlands; ^4^ Plant Production Systems Group Wageningen University P.O. Box 430 6700 AK Wageningen The Netherlands; ^5^ Centre for Crop Systems Analysis Wageningen University P.O. Box 430 6700 AK Wageningen The Netherlands; ^6^ School of Geography University of Leeds Woodhouse Lane Leeds LS2 9JT UK; ^7^ Department of Geography University College London Gower Street London WC1E 6BT UK; ^8^ Mensuration Unit Forestry Commission of Ghana Kumasi Ghana; ^9^ Escuela ECAPMA, UNAD Calle 14 Sur No. 14‐23 Bogotá Colombia; ^10^ Fundación Con Vida Avenida del Río # 20‐114 Medellín Colombia; ^11^ Projeto Dinâmica Biológica de Fragmentos Florestais Instituto Nacional de Pesquisas da Amazônia - INPA Av. André Araújo 2936 Manaus Amazonas 69067-375 Brazil; ^12^ Remote Sensing Division National Institute for Space Research - INPE Av. dos Astronautas 1758 São José dos Campos São Paulo 12227-010 Brazil; ^13^ Geography, College of Life and Environmental Sciences University of Exeter North Park Road Exeter EX4 4QE UK; ^14^ Museo de Historia Natural Noel Kempff Mercado Universidad Autónoma Gabriel Rene Moreno Avenida Irala 565, Casilla Postal 2489 Santa Cruz Bolivia; ^15^ Naturalis Biodiversity Center Darwinweg 2 2332 CR Leiden The Netherlands; ^16^ Inventory & Monitoring Program National Park Service 120 Chatham Lane Fredericksburg 22405 Virginia USA; ^17^ Center for Conservation and Sustainability Smithsonian Conservation Biology Institute 1100 Jefferson Dr. SW, Suite 3123 Washington 20560-0705 D.C. USA; ^18^ Department of Botany & Plant Physiology Faculty of Science University of Buea P.O. Box 063 Buea Cameroon; ^19^ School of Geography, Earth and Environmental Sciences University of Plymouth Plymouth PL4 8AA UK; ^20^ Centre for Tropical Environmental and Sustainability Science and College of Science and Engineering James Cook University 14-88 McGregor Road Cairns 4878 Australia; ^21^ Department of Environmental Science and Policy George Mason University Fairfax Virginia USA; ^22^ Environmental Change Institute School of Geography and the Environment, University of Oxford Oxford OX13QY UK; ^23^ Universidade do Estado de Mato Grosso Av. Prof. Dr. Renato Figueiro Varella, s/n, Bairro Olaria Nova Xavantina State of Mato Grosso CEP 78690-000 Brazil; ^24^ Tropical Forests and People Research Centre University of the Sunshine Coast Queensland 4556 Australia; ^25^ Department of Environment and Geography University of York York YO10 5NG UK; ^26^ Flamingo Land Ltd. Malton, North Yorkshire YO17 6UX UK; ^27^ Facultad de Ingeniería Ambiental Universidad Estatal Amazónica Puyo, Pastaza Ecuador; ^28^ Herbario Vargas Universidad Nacional de San Antonio Abad del Cusco Avenida de la Cultura, Nro 733 Cusco Peru; ^29^ Science and Education The Field Museum 1400S. Lake Shore Drive Chicago 60605-2496 Illinois USA; ^30^ Center for Tropical Conservation Nicholas School of the Environment, Duke University P.O. Box 90381 Durham 27708 North Carolina USA; ^31^ Bureau Waardenburg P.O. Box 365 4100 AJ Culemborg The Netherlands; ^32^ Museu Universitário Universidade Federal do Acre Acre Brazil; ^33^ Plant Systematic and Ecology Laboratory University of Yaounde I Yaounde Cameroon; ^34^ Centre for International Forestry Research (CIFOR) Jalan CIFOR, Situ Gede, Sindang Barang Bogor 16115 Indonesia; ^35^ Forest Sciences Centre University of British Columbia 2424 Main Mall Vancouver V6T 1Z4 British Columbia Canada; ^36^ Systems Ecology Vrije Universiteit De Boelelaan 1087 1081 HV Amsterdam The Netherlands; ^37^ Department of Biology and Florida Museum of Natural History University of Florida Gainesville 32611 Florida USA; ^38^ School of Geography University of Nottingham University Park Nottingham NG7 2RD UK; ^39^ Instituto de Investigaciones para el Desarrollo Forestal Universidad de Los Andes Mérida Venezuela; ^40^ Universidad Autónoma de Beni Riberalta, Beni Bolivia; ^41^ Agence Nationale des Parcs Nationaux Libreville BP 20379 Gabon; ^42^ Institut de Recherche en Ecologie Tropicale Libreville BP 13354 Gabon; ^43^ School of Natural Sciences University of Stirling Stirling FK9 4LA UK; ^44^ School of Natural Sciences Bangor University Bangor, Gwynedd LL57 2DG UK

**Keywords:** climatic water deficit, competition, forest dynamics, mortality, neighborhood effects, soil fertility, trait‐based models, tree growth, tropical forest, wood density

## Abstract

Competition among trees is an important driver of community structure and dynamics in tropical forests. Neighboring trees may impact an individual tree’s growth rate and probability of mortality, but large‐scale geographic and environmental variation in these competitive effects has yet to be evaluated across the tropical forest biome. We quantified effects of competition on tree‐level basal area growth and mortality for trees ≥10‐cm diameter across 151 ~1‐ha plots in mature tropical forests in Amazonia and tropical Africa by developing nonlinear models that accounted for wood density, tree size, and neighborhood crowding. Using these models, we assessed how water availability (i.e., climatic water deficit) and soil fertility influenced the predicted plot‐level strength of competition (i.e., the extent to which growth is reduced, or mortality is increased, by competition across all individual trees). On both continents, tree basal area growth decreased with wood density and increased with tree size. Growth decreased with neighborhood crowding, which suggests that competition is important. Tree mortality decreased with wood density and generally increased with tree size, but was apparently unaffected by neighborhood crowding. Across plots, variation in the plot‐level strength of competition was most strongly related to plot basal area (i.e., the sum of the basal area of all trees in a plot), with greater reductions in growth occurring in forests with high basal area, but in Amazonia, the strength of competition also varied with plot‐level wood density. In Amazonia, the strength of competition increased with water availability because of the greater basal area of wetter forests, but was only weakly related to soil fertility. In Africa, competition was weakly related to soil fertility and invariant across the shorter water availability gradient. Overall, our results suggest that competition influences the structure and dynamics of tropical forests primarily through effects on individual tree growth rather than mortality and that the strength of competition largely depends on environment‐mediated variation in basal area.

## Introduction

Competition is an important driver of community structure and dynamics in forests worldwide (Kunstler et al. 2016), particularly in closed‐canopy forests such as mature, undisturbed tropical forests, where low light levels under the canopy typically limit tree growth. Generally, competition with neighboring trees is expected to decrease growth and increase the probability of mortality of individual tropical trees (Uriarte et al. 2004, Lasky et al. 2015). However, effects of competition on growth and mortality of individual trees have only been quantified within single tropical forest sites to date (e.g., Uriarte et al. 2004, Baribault et al. 2012). Whether strong effects of competition on demographic rates are pervasive, and whether they vary across environmental gradients in the tropics, remains unresolved.

Better knowledge of the effects of competition on tropical tree growth and mortality, and the geographic variation thereof, is essential for enhancing understanding of the global terrestrial carbon balance. Mature tropical forests have increased in biomass over recent decades (Lewis et al. 2009), and those in Amazonia have become more dynamic (McDowell et al. 2018). Mortality rates have a key role in controlling biomass in tropical forests (Johnson et al. 2016), as increases in mortality over time are influencing the carbon balance of Amazon forests (Brienen et al. 2015). Changes in the average strength of competition in forests might be one of the driving factors of such dynamic changes, because increased biomass (i.e., increased neighborhood crowding) leads to enhanced competition, with expected impacts in turn in decreased growth and increased mortality. More generally, the underlying causes of tree mortality in the tropics are still actively debated (e.g., McDowell et al. 2018), and quantifying their effects on the terrestrial carbon balance is a key challenge for ecologists and global change scientists. In addition to mortality that results from competition, trees may die from a range of other processes, including hydraulic failure in response to drought (large trees in particular; Phillips et al. 2010, Bennett et al. 2015, Rowland et al. 2015), from senescence (although effects are weak; Mencuccini et al. 2005), and from large‐scale wind disturbance (Espírito‐Santo et al. 2014), but which process(es) dominate(s) remains poorly understood.

Environmental conditions vary considerably across tropical forest sites, and this variation is known to influence forest structure and dynamics strongly. Across the Amazon basin, for example, water availability generally decreases from north to south, and soil fertility increases from east to west (ter Steege et al. 2006). Drier forests generally have a lower stature, lower aboveground biomass and basal area, and a more open canopy than wet forests (Quesada et al. 2012), with typically lower rates of tree growth (Toledo et al. 2011) and stem turnover (Quesada et al. 2012). Forests are more dynamic on the high‐fertility soils of western Amazonia, with higher coarse woody productivity (Malhi et al. 2004, Baker et al. 2009), higher stem mortality (Johnson et al. 2016), lower basal area and aboveground biomass, and lower mean wood density (WD) than eastern Amazonia (Baker et al. 2004, Malhi et al. 2006, ter Steege et al. 2006, Quesada et al. 2012). Environmental gradients are also found across African tropical forests, where basal area decreases with both rainfall seasonality and soil fertility (sum of bases; Lewis et al. 2013).

Effects of competition on tree growth and mortality are expected to vary across continental environmental gradients in Amazonia and tropical Africa because water and soil nutrient availability influence forest structure and understory light availability. Competition has been hypothesized to intensify with resource availability because high resource levels lead to rapid growth and resource depletion, whereas plant growth is generally low in stressful habitats (Grime 1979). In tropical forests, competition is likely to be strongest at high resource (water and/or soil nutrient availability) levels, which support a higher basal area. Then, the resulting crowding leads to stronger competition because of reduced light availability to individual trees.

The response of any given focal tree to competition will likely depend not only on the degree of crowding in its local neighborhood, but also on its size and functional traits. Smaller trees are more strongly affected by competition (Uriarte et al. 2004) because they are more heavily shaded by taller neighbors, and likely suffer from greater belowground competition. Shade‐intolerant tree species, which typically have low wood density (WD; van Gelder et al. 2006), respond more strongly to changes in light availability than shade‐tolerant species (Bazzaz 1979), and thus are likely to be more strongly affected by competition. Indeed, shade‐intolerant (Hubbell et al. 2001, Canham et al. 2006, Kunstler et al. 2011) and low WD tree species (Kunstler et al. 2016) often show greater growth decreases in response to neighborhood crowding. Hence, variation in the plot‐level strength of competition (i.e., the extent to which growth is reduced, or mortality is increased, by competition across all individual trees in a plot) across environmental gradients may not only depend on forest basal area, but also on tree size distributions and mean wood density. Nevertheless, forest basal area is expected to have the largest effect, because the basal area of neighbor trees directly influences resource availability to a focal tree.

In this study, we quantify the effects of neighborhood crowding on tree growth and mortality across gradients of moisture and soil nutrient availability in Amazonia and tropical Africa. Neighborhood crowding likely reflects competition for light (although competition for water and soil nutrients may also play a role), as light is typically the main factor limiting tree growth in closed‐canopy forests. We use data from 151 ~1 ha‐plots to fit nonlinear growth and mortality models based on tree WD, size, and neighborhood crowding. We use these models to estimate the predicted plot‐level strength of competition, i.e., to what extent growth across all trees is reduced compared to a low level of neighborhood crowding, and assess how water availability and soil fertility influence the strength of competition through relationships with average tree size, plot basal area, and plot wood density. Specifically, we test the following predictions: (1) tree growth will decrease, and mortality increase, with neighborhood crowding; (2) low WD species will be most strongly affected by neighborhood crowding; (3) variation in the plot‐level strength of competition will be more strongly related to plot basal area than to wood density or mean tree size; (4) the plot‐level strength of competition will intensify with increasing climatic water availability through relationships with plot basal area on both continents; and (5) the predicted plot‐level strength of competition will be negatively related to soil fertility in Africa because of decreasing basal area with increasing soil fertility (sum of bases; Lewis et al. 2013), but be largely independent of soil fertility in Amazonia because of weak correlations between soil fertility and basal area (Quesada et al. 2012).

## Methods

### Plot data

**Fig. 1:**
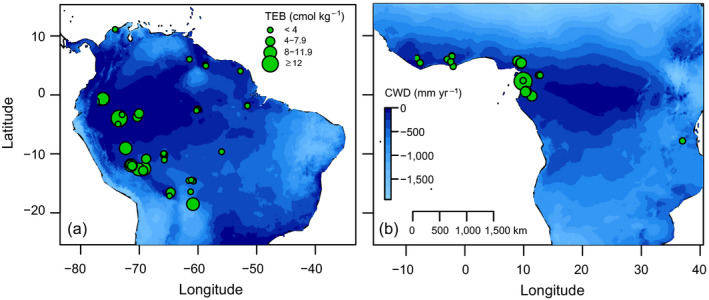
Maps of the plot locations across gradients in climatic water deficit (CWD) and soil total exchange bases (TEB). (a) Amazonia (102 plots); (b) tropical Africa (49 plots).

We used data from 102 permanent plots in Amazonia from the RAINFOR network and 49 in tropical Africa from the AfriTRON network, curated at ForestPlots.net (Lopez‐Gonzalez et al. 2009, 2011; Fig. 1), to span the environmental gradients in each tropical lowland forest region. Plots were all below 500 m above sea level (a.s.l.), in nonflooded, closed‐canopy forests, with a fivefold range of mean annual precipitation in Amazonia (855–4273 mm) and twofold range in Africa (1,377–2,716 mm). Soil fertility, estimated by soil total exchange bases (in cmol(+)/kg), varied from 0.5 to 13.2 cmol(+)/kg in Amazonia, and from 2 to 13.5 cmol(+)/kg in Africa. Most plots were 1 ha in size, but plot size ranged from 0.25 to 9 ha (Appendix S1: Table S1). Trees ≥10‐cm diameter at breast height (dbh), or above buttresses, were measured for their diameter, identified to species, and either mapped or assigned to 0.04‐ha subplots. Across all plots, 2,947 species and 73,100 trees were included in Amazonia, and 695 species and 20,705 trees in Africa. For each plot, we included data from two censuses with an average interval length of 6.3 yr (range: 3.0–12.7 yr; Appendix S1: Table S1) and an average starting year of 1994 (range: 1971–2008), and calculated annual basal area growth (in cm^2^/yr) for trees that were present in both censuses. We excluded monocotyledonous species (palms and Strelitziaceae) from the growth models, as they do not have secondary growth. Neighborhood crowding was expressed as the total basal area of neighbor trees within a 0.04‐ha subplot (BA_neigh_) in the first census. We defined neighborhoods based on subplots instead of on a fixed radius around each focal tree, to allow inclusion of plots for which individual trees were not mapped (Appendix S2). We found that BA_neigh_ accurately captured local effects of competition (Appendix S2). Neighborhood crowding likely reflects competition for light, although competition for water and soil nutrients may also occur. Other processes, for example pathogen accumulation at high densities of conspecific trees that increase mortality (negative density dependence; NDD), may also contribute, but effects of NDD are typically weak for large trees (Zhu et al. 2015).

### Environmental conditions and wood density

Average annual rainfall (in mm/yr) for each of the plots was obtained from WorldClim 2 (Fick and Hijmans 2017). Climatic water deficit (CWD; in mm/yr; Chave et al. 2014) was included as a measure of seasonal drought stress.^1^ CWD is defined as the cumulative amount of water lost by the environment during months in which evapotranspiration exceeds rainfall. CWD is negative for sites that experience seasonal drought stress; a CWD of 0 indicates absence of seasonal drought stress. Topsoil total exchange bases (TEB; in cmol(+)/kg) was included as an indicator of soil fertility, and was obtained from the World Harmonized Soil Database (FAO/IIASA/ISRIC/ISS‐CAS/JRC 2012). Wood density (WD) data were obtained from a global database (Chave et al. 2009, Zanne et al. 2009). In cases where a species‐specific WD value was not available, we used genus‐ or family‐level mean WD (Baker et al. 2004). Genus‐level WD was used for 1,578 (out of 2,947) and for 233 (out of 695) species in Amazonia and Africa, respectively. Family‐level WD was used for 235 and 186 species in Amazonia and Africa, respectively. For stems that remained unidentified, or for which family‐level mean WD was unavailable (for 37 species in Amazonia and 31 in Africa), we used the mean WD across all stems in the plot.

### Modeling approach

We used a combination of (modeling) approaches to evaluate whether the predicted strength of competition varied across environmental gradients in Amazonia and Africa. First, we used the plot data from both continents to construct nonlinear models of individual tree growth and mortality as functions of tree size (dbh), neighborhood crowding, and WD. Separate models were fitted for Amazonia and tropical Africa. Second, we used the estimated parameters of the fitted growth models to calculate the strength of competition (*C*_plot_) at the plot level (mortality was excluded because competition effects on mortality were very weak; see Results). As a last step, we assessed (1) whether *C*_plot_ varied with water availability and soil fertility, and (2) how *C*_plot_ was influenced by variation in plot basal area, plot‐level WD, and average tree size. Variation in *C*_plot_ could arise from plot‐to‐plot differences in average neighborhood crowding (i.e., plot basal area), average WD, or average tree size, as each of these influenced the modeled effect of competition on individual tree growth. We describe each of these steps in greater detail below.

We modeled the annual basal area growth (*G*) and the annual probability of mortality (*M*) for individual trees on each continent as follows:

$$G=a_{G}\times p_{G}\times S_{G}\times C_{G}$$

$$M={\left(1+a_{M}\times p_{M}\times S_{M}\times C_{M}\right)}^{-1}$$

where *a_G_* and *a_M_* are constants, *p_G_* and *p_M_* are plot‐level random effects, and *S* and *C* (each subscripted for growth and mortality) are nonlinear functions that capture effects of tree size and competition, respectively:

$$S={\text{dbh}}^{s_{1}}\times \exp\left(-s_{2}\times \text{dbh}\right)$$

$$C=\exp\left(-c_{1}\times {\text{dbh}}^{c_{2}}\times {\text{BA}}_{\text{neigh}}\right)$$

where *s*_1_, *s*_2_, *c*_1_, and *c*_2_ control the shape of the functions and have separate values for growth and mortality. *S* has a flexible form that can produce either an intermediate peak or a continuous increase in tree growth with tree size (dbh; Coomes et al. 2014). For mortality, *S* can produce a U‐shaped response where mortality both decreases with size for small trees and increases with size for larger trees (Rüger et al. 2011, Iida et al. 2014). *C* is a decreasing function that can produce lower growth and higher mortality in trees with greater neighborhood crowding. The sensitivity of growth and mortality to competition may vary with tree size (as determined by *c*_2_), as large trees may be less susceptible to competition than small trees.

We applied a trait‐based approach to account for taxonomic variation in growth and mortality, as a species‐level approach was not feasible given the huge diversity of tree species in the tropics (e.g., an estimated 15,000 tree species in the Amazon basin; ter Steege et al. 2015). WD is known to be a good predictor of tropical tree growth and mortality (e.g., Chao et al. 2008, Poorter et al. 2008, Wright et al. 2010, Rüger et al. 2012, Aleixo et al. 2019); therefore we defined model parameters *a*, *s*_1_, *s*_2_, *c*_1_, and *c*_2_ as linear functions of WD. As such, WD could influence growth and mortality directly, as well as indirectly through effects on size relationships and responses to competition (e.g., Hérault et al. 2011, Iida et al. 2014, Kunstler et al. 2016). Models were fit using a hierarchical Bayesian approach (Appendix S2, Data S1: Model_script.R).

Using the fitted growth models, we calculated the strength of competition for each plot (*C*_plot_) as the percent reduction in plot‐level basal area growth due to competition compared to a low, baseline level of neighborhood crowding by assessing to what extent growth was reduced for each individual tree:

$$C_{\text{plot}}=\left(1-\left(\frac{\sum_{i=1}^{n}G_{i}^{〈c〉}}{\sum_{i=1}^{n}G_{i}^{〈\mathrm{lc}〉}}\right)\right)\times 100$$

where for tree *i*, $G_{i}^{〈c〉}$ represents predicted basal area growth with the observed level of competition, and $G_{i}^{〈\mathrm{lc}〉}$ represents its potential growth at a low, baseline level of competition. Quantifying plot‐level competition based on the growth reduction compared to potential growth in the absence of competition may be unrealistic, because a BA_neigh_ of zero is rarely found. Per continent, we calculated the 10th percentile of the plot‐level 10th percentile values of BA_neigh_ (11.3 m^2^/ha for Amazonia; 9.8 m^2^/ha for Africa). We therefore calculated the strength of competition based on a general baseline level of BA_neigh_ = 10 m^2^/ha for both continents. Thus, *C*_plot_ was calculated by comparing predicted plot‐level growth (based on all individual trees) with competition to growth at a BA_neigh_ of 10 m^2^/ha. Growth predictions were based on the posterior means of the model parameters.

For each continent, we examined whether *C*_plot_ was correlated with water availability (CWD) or soil fertility (TEB). To assess whether variation in *C*_plot_ was driven by variation in plot basal area, plot‐level WD (basal area‐weighted mean), or average tree size (the diameter of a tree with mean basal area; $\sqrt{\left(\sum {\text{dbh}}^{2}\right)/n}$)), we modeled *C*_plot_ as a function of plot BA, plot‐level WD, and average tree size using linear regression. In order to compare effect sizes among the three predictors, predictors were standardized by subtracting the mean and dividing the difference by the standard deviation. All analyses were performed in R 3.1.2 (R Development Core Team 2014).

## Results

### Overall responses to competition

**Fig. 2:**
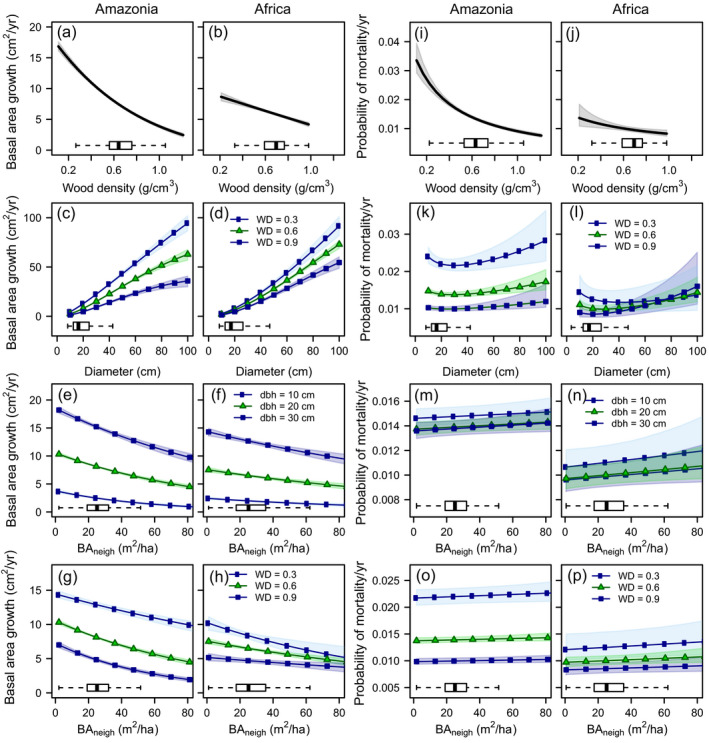
Effects of wood density (WD), tree size, and competition (subplot neighbor basal area; BA_neigh_) on predicted annual basal area growth and mortality across Amazonia (*n* = 102 plots) and tropical Africa (*n* = 49). Solid lines and symbols indicate predicted effects based on the posterior means; shaded areas indicate the 95% credible interval. Boxplots indicate the distribution of the variable on the *x*‐axis. BA_neigh_ was kept constant at the mean for quantifying effects of WD and tree size on growth and mortality; tree size was kept constant at 20‐cm diameter for quantifying effects of WD and BA_neigh_. dbh = diameter at breast height.

**Fig. 3:**
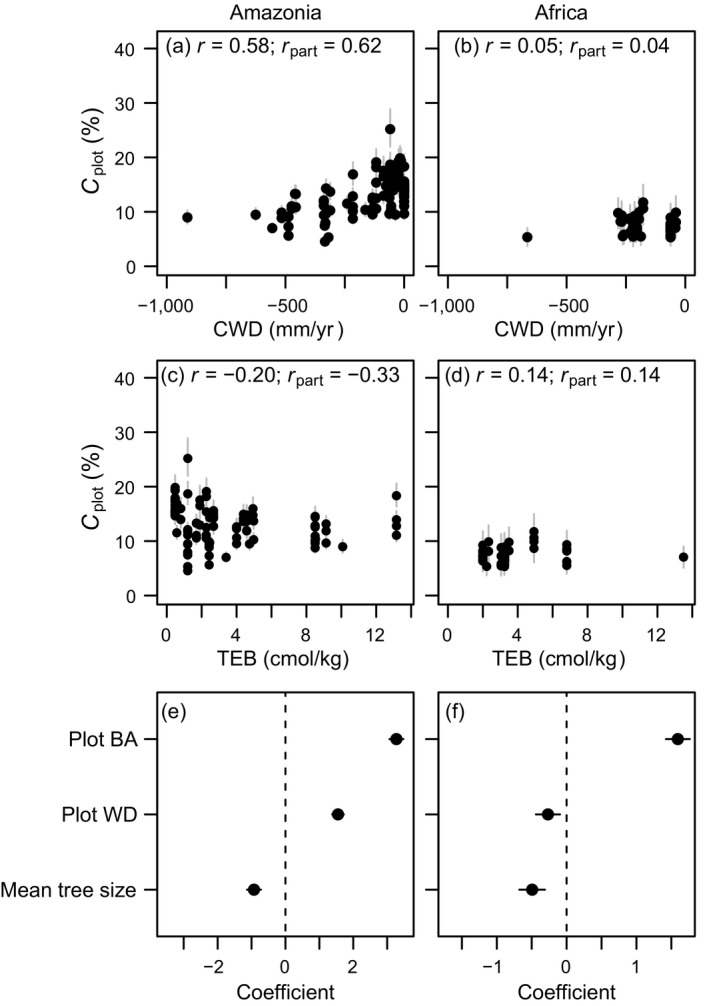
Relationships between the strength of competition on basal area growth (*C*_plot_: reduction in plot‐level basal area growth by competition based on a reference value of 10 m^2^/ha) and climatic water deficit (CWD), soil total exchange bases (TEB), plot basal area (BA), plot wood density (WD), and mean tree size in Amazonia (*n* = 102 plots) and tropical Africa (*n* = 49 plots). (a–d) Gray bars represent 95% credible intervals; Pearson’s correlation (*r*) and partial (*r*_part_) correlation coefficients are indicated; (e, f) standardized regression coefficients with 95% confidence intervals are indicated.

Individual tree growth was strongly affected by competition (Fig. 2; Appendix S1: Table S2), but competition effects were stronger in Amazonian than African tropical forests. For example, for a 20‐cm diameter tree with a WD of 0.6 g cm^−3^, growth decreased by 34% in Amazonia (Fig. 2e, g) and 17% in Africa (Fig. 2f, h) as BA_neigh_ increased from 10 to 50 m^2^/ha. Further, even though plot‐level basal area was on average slightly lower in Amazonia (25.9 ± 0.44 m^2^/ha; mean ± SE) than in Africa (28.7 ± 0.64 m^2^/ha), the stronger response of trees to competition in Amazonia resulted in greater predicted decreases in plot‐level wood production than in Africa. Competition reduced plot‐level basal area growth (compared to a baseline, low‐BA_neigh_ value of 10 m^2^/ha) by, on average, 31.1% (range: 4.5–25.2%; Fig. 3a, c) in Amazonia, and by 7.4% in Africa (range: 5.3–11.7%; Fig. 3b, d).

In contrast to effects on growth, competition with neighboring trees had little or no effect on the probability of mortality. Nevertheless, the mortality model that included competition performed better than the no‐competition model for Amazonia (Appendix S1: Table S2). The predicted probability of mortality for a 20‐cm dbh tree with a WD of 0.6 gr cm^−3^ remained constant at 1.4% (Fig. 2m, o) and 1.0% (Fig. 2n, p) per year as BA_neigh_ increased from 10 to 50 m^2^ ha^−1^ in Amazonia and Africa, respectively.

### Effects of wood density and tree size

Tree basal area growth decreased with increasing WD on both continents (Fig. 2a, b). In Amazonia, a 20‐cm tree with low WD (0.3 g/cm^3^) grew more than twice as fast as a high‐WD (0.9 g/cm^3^) tree of the same size (Fig. 2a). In Africa, the growth decrease with increasing WD was less pronounced (Fig. 2b). Growth increased with tree size on both continents (Fig. 2c, d), with low WD species exhibiting stronger size‐related increases in growth.

On both continents, small trees were more strongly affected by competition than large trees (Fig. 2e, f). In Amazonia, growth of a 10‐cm tree and a 30‐cm tree decreased by 49% and 27%, respectively, as neighbor basal area increased from 10 to 50 m^2^/ha (Fig. 2e). Similar growth decreases were found in Africa, with a 28% and 18% growth decrease for a 10‐cm and a 30‐cm tree, respectively (Fig. 2f). Amazonian trees with different WD showed similar absolute decreases in growth resulting from competition, but on a proportional basis high WD species expressed greater decreases than low WD species (48% and 17%, respectively) as BA_neigh_ increased from 10 to 50 m^2^/ha (Fig. 2g). Conversely, the growth of high WD species in Africa was less affected by competition than that of low WD species (decreases of 14% and 28%, respectively; Fig. 2h).

The probability of mortality decreased with WD on both continents (Fig. 2i, j), but the decline was more pronounced and more consistent in Amazonia than in Africa. Mortality generally increased with tree size (Fig. 2k, l), particularly for trees >50‐cm dbh, although low abundances increased uncertainty for large trees. Small trees with low WD had higher mortality than mid‐sized trees (7% and 23% higher mortality at 10‐cm dbh than at 50‐cm dbh in Amazonia and tropical Africa, respectively), leading to a U‐shaped size–mortality relationship. Effects of competition on mortality were very weak on both continents, regardless of WD or tree size (Fig. 2m, n, o, p).

### Variation in the strength of competition

In Amazonia, the plot‐level strength of competition (*C*_plot_) was strongly and positively correlated with CWD, but negatively correlated with TEB, particularly after accounting for variation in CWD (Fig. 3a, b). Plot basal area had the largest effect on *C*_plot_, followed by a positive effect of plot WD, and a small negative effect of mean tree size (Fig. 3e). In Africa, *C*_plot_ was not correlated with CWD, and just weakly, positively correlated with TEB (Fig. 3c, d). Like in Amazonia, *C*_plot_ was largely driven by a positive effect of plot basal area. Unlike Amazonia, plot‐level WD had little influence on *C*_plot_ in tropical Africa (Fig. 3f).

## Discussion

### Large variation in the strength of competition on tree growth across environmental gradients

Across two continents, we found that competition is an important driver of tropical tree growth, but unexpectedly not of mortality. Variation in the plot‐level strength of competition across tropical forests was large for both continents. As expected, individual tree growth was most strongly affected by competition in forests with high basal area, although in Amazonia competition was also strong in high WD forests. In Amazonia, as expected, the strength of competition on tree growth increased with water availability (CWD), likely because of higher plot basal area in wetter forests (Appendix S1: Fig. S1). However, the strength of competition declined slightly with soil fertility (TEB), likely because of lower plot‐level WD at high soil fertility (Appendix S1: Table S3), and because low WD species in Amazonia appeared to be less susceptible to competition. Unexpectedly, the strength of competition did not vary with water availability, nor with soil fertility, in Africa. This may have been due to the shorter water availability and soil fertility gradients compared to Amazonia in our study, which likely partly explains the lack of relationships with environmental conditions in tropical Africa. Given these differences, we must be careful in drawing general conclusions across continents. Across the same range in environmental conditions (based on Africa, excluding two outliers; Fig. 3b, d), the relationship between the strength of competition and CWD was stronger in Amazonia (Pearson’s *r* = 0.40, *n* = 38 plots) than in Africa (*r* = −0.12). The relationship between the strength of competition and TEB was somewhat stronger for Africa because of outlier exclusion (*r* = 0.23) than for Amazonia (*r* = 0.10, *n* = 41 plots). Overall, our results are partly consistent with Grime’s (1979) hypothesis that competition is strongest in resource‐rich environments because of the increased strength of competition under high water availability in Amazonia.

### Effects of WD and tree size on growth and mortality

In contrast, effects of WD and tree size on individual tree growth and mortality were largely consistent between Amazonia and tropical Africa. In general, our results confirmed findings of previous studies that were based on a single, or a few, tropical forest sites, and indicated that these attributes control growth and mortality across most of the tropical forest biome. Tree growth and mortality both decreased with WD, as reported by smaller‐scale Neotropical studies (e.g., Chao et al. 2008, Keeling et al. 2008, Poorter et al. 2008, Wright et al. 2010, Rüger et al. 2012). Low WD is associated with an acquisitive strategy that confers rapid growth, but that comes at the cost of high mortality because of lower tolerance to stress and damage compared to high WD species (Wright et al. 2010). Basal area growth increased with tree size, presumably because larger trees have more resources and/or leaf area available to support assimilation of carbon (Stephenson et al. 2014). The ontogenetic increase in growth was strongest for low WD species (Fig. 2c, d), probably because of the low construction cost of low‐density wood. These findings are consistent with single‐site studies that found that low‐WD tropical tree species had the strongest increase in diameter growth at intermediate tree size (King et al. 2006, Hérault et al. 2011, but see Rüger et al. 2012).

Our study is one of the first to show a clearly U‐shaped size–mortality relationship (cf. Rüger et al. 2011, Iida et al. 2014, Pillet et al. 2018), which we found for low‐WD species. For trees ≥30 cm dbh, and for high WD trees in general, the risk of death increased nearly monotonically with size. Small trees, particularly those with low WD, may be most susceptible to physical damage in the understory (Clark and Clark 1991). The higher mortality risk for large trees may be a result of the stronger risk of hydraulic failure for large trees (Rowland et al. 2015) rather than senescence (Mencuccini et al. 2005).

### Competition decreased tree growth but did not influence mortality

Our results show that growth decreases with increased neighborhood crowding across tropical forests on two continents, particularly for small trees. This provides large‐scale confirmation that results reported to date for single Neotropical forest sites in Costa Rica, Ecuador, Panama, and Puerto Rico (Uriarte et al. 2004, Baribault et al. 2012, Grote et al. 2013, Lasky et al. 2015, Fortunel et al. 2016) are typical of the biome. We also expected that low‐WD species would be most strongly affected by competition. Low‐WD species were indeed most affected by competition in Africa, consistent with earlier findings of strong growth responses of low‐WD species to competition (Kunstler et al. 2016) and light availability (Rüger et al. 2012), which supports the notion that shade‐intolerant tree species respond more strongly to changes in resource levels. However, it remains unclear why high WD species in Amazonia were more susceptible to competition. The mean and range of neighborhood crowding levels did not vary across WD classes (<0.35 g/cm^3^; 0.35–0.75 g/cm^3^; >0.75 g/cm^3^; results not shown), thus effects of competition were not weaker because low WD species were confined to areas with low neighborhood crowding.

Our results suggest that competition does not strongly influence tree mortality in either Amazonia or tropical Africa. The lack of evidence for impacts of competition on mortality could be partly due to only including trees ≥10 cm dbh in our study. Generally, mortality rates are highest for seedlings and saplings (trees <10 cm dbh; Clark and Clark 1992, Condit et al. 1995) because of the low‐light conditions in the understory, and mortality resulting from negative density‐dependent effects (Zhu et al. 2015). Those studies that have found clear effects of competition on tropical tree mortality included trees <10 cm dbh, and likely included a larger range of resource levels by focusing on forests in recovery from disturbances such as agricultural use (Lasky et al. 2014) and hurricanes (Uriarte et al. 2004). Our findings suggest that competition is not a widespread and important driver of mortality for trees ≥10 cm dbh in mature tropical forests. Instead, it appears that processes such as hydraulic failure (e.g., Rowland et al. 2015) and stochastic wind disturbances (Espírito‐Santo et al. 2014, Aleixo et al. 2019) may be the dominant causes of mortality, although accelerated growth may eventually increase mortality by ensuring that trees reach larger sizes more quickly (cf. Brienen et al. 2015, McDowell et al. 2018). Nevertheless, the effects of competition on growth may still indirectly lead to an increased risk of mortality, as suppressed trees will be less likely to escape from suppression because of their slow growth, and thus accumulate mortality risk over a longer period of time.

### Implications for projecting the tropical forest carbon sink

Our results provide some insights into how competition may influence ongoing and future changes in the tropical forest carbon sink. First, we found that the decrease in basal area growth due to competition increased strongly with forest basal area. Hence, when forests gain basal area over time, greater competition between trees is likely to reduce tree growth, which might explain why long‐term increases in productivity in Amazonia have leveled off since 2000 (Brienen et al. 2015). Secondly, we found that, particularly in Amazonia, effects of competition are also influenced by stand‐level WD. Changes in WD over time (e.g., van der Sande et al. 2016) may not only influence standing biomass (Baker et al. 2004), but also alter the strength of competition.

Competition effects should be appropriately incorporated into models that are used for projecting future dynamics of tropical forests. In individual‐based forest dynamics models, effects of competition are typically included (Fyllas et al. 2014), but models could be further improved by also including effects of WD, and tree size, on the strength of competition. These changes are relatively easy to implement, as direct effects of tree size are already included, and WD data are available for many species (Chave et al. 2009). In dynamic global vegetation models that are applied over broad geographical scales, inclusion of forest basal area as a measure of neighborhood crowding will mostly account for geographical variation in the strength of competition. Such models could be improved further by including average plot WD.

In conclusion, our study revealed that in 151 forest plots distributed across Amazonia and tropical Africa competition is an important driver of individual tree growth rates, but not of the probability of tree mortality. This is, to our knowledge, the first study to evaluate the effects of competition on tropical tree growth and mortality at such a broad geographical scale. Given that geographic variation in the strength of competition is mainly driven by forest basal area (i.e., neighborhood crowding), we anticipate that wood production might decrease as tropical forests accrue higher basal area.

## Supporting information

Appendix S1

Appendix S2

Metadata S1

Data S1

## Footnotes

^1^

http://chave.ups‐tlse.fr/pantropical_allometry.htm
