# Supplementary material for: Competition influences tree growth, but not mortality, across environmental gradients in Amazonia and tropical Africa
Source: Ecology. 2020 May 5;101(7):e03052. doi: 10.1002/ecy.3052 (PMC7379300; doi:10.1002/ecy.3052)
Supplement: Supplementary file 1 — Appendix S1 [file ECY-101-e03052-s003.pdf]

Supporting Information. Rozendaal, D.M.A., O.L. Phillips, S.L. Lewis, K. Affum-Baffoe, E. Alvarez Dávila, A. Andrade, L.E.O.C. Aragão, A. Araujo-Murakami, T.R. Baker, O. Bánki, R.J.W. Brienen, J.L.C. Camargo, J.A. Comiskey, M.N. Djuikouo K., S. Fauset, T.R. Feldpausch, T.J. Killeen, W.F. Laurance, S.G.W. Laurance, T. Lovejoy, Y. Malhi, B.S. Marimon, B.-H. Marimon Junior, A.R. Marshall, D.A. Neill, P. Núñez Vargas, N.C.A. Pitman, L. Poorter, J. Reitsma, M. Silveira, B. Sonké, T. Sunderland, H. Taedoumg, H. ter Steege, J.W. Terborgh, R.K. Umetsu, G.M.F. van der Heijden, E. Vilanova, V. Vos, L.J.T. White, S. Willcock, L. Zemagho, and M.C. Vanderwel. 2020. Competition influences tree growth, but not mortality, across environmental gradients in Amazonia and tropical Africa. Ecology.

Appendix S1

Table S1. List of included plots.

Continent	Country	Plot name	Plot code	Trees mapped / subplots	Plot area (ha)	Latitude	Longitude
Amazon	Bolivia	BEEM plot 5	BEE-05	mapped	1	-16.5	-64.6
Amazon	Bolivia	Chore 1	CHO-01	0.04 ha subplots	1	-14.4	-61.1
Amazon	Bolivia	Cerro Pelao 1	CRP-01	0.04 ha subplots	1	-14.5	-61.5
Amazon	Bolivia	Cerro Pelao 2	CRP-02	mapped	1	-14.5	-61.5
Amazon	Bolivia	Huanchaca Dos, plot 1	HCC-21	0.04 ha subplots	1	-14.5	-60.7
Amazon	Bolivia	Huanchaca Dos, plot 2	HCC-22	0.04 ha subplots	1	-14.5	-60.7
Amazon	Bolivia	Isla Huanchaca, plot 1	HCC-23	0.04 ha subplots	1	-14.6	-60.7
Amazon	Bolivia	Los Fierros Bosque I	LFB-01	0.04 ha subplots	1	-14.6	-60.8
Amazon	Bolivia	Los Fierros Bosque II	LFB-02	0.04 ha subplots	1	-14.6	-60.8
Amazon	Bolivia	Las Londras, plot 1	LSL-01	0.04 ha subplots	1	-14.4	-61.1
Amazon	Bolivia	Las Londras, plot 2	LSL-02	0.04 ha subplots	1	-14.4	-61.1
Amazon	Bolivia	Mabet plot 1	MBT-01	mapped	1	-10.1	-65.9
Amazon	Bolivia	Mabet plot 4	MBT-04	mapped	1	-10.3	-65.6
Amazon	Bolivia	Mabet plot 5	MBT-05	mapped	1	-10.0	-65.6
Amazon	Bolivia	Mabet plot 6	MBT-06	mapped	1	-10.0	-65.6
Amazon	Bolivia	Mabet plot 7	MBT-07	mapped	1	-9.9	-65.7
Amazon	Bolivia	Mabet plot 8	MBT-08	mapped	1	-9.9	-65.8
Amazon	Bolivia	Ottavio Ranch, Bolivia, plot 1, forest	OTT-01	mapped	1	-16.4	-61.2
Amazon	Bolivia	Reserva El Tigre 05	RET-05	mapped	1	-11.0	-65.7
Amazon	Bolivia	Reserva El Tigre 06	RET-06	mapped	1	-11.0	-65.7
Amazon	Bolivia	Reserva El Tigre 08	RET-08	mapped	1	-11.0	-65.7
Amazon	Bolivia	Reserva El Tigre 09	RET-09	mapped	1	-11.0	-65.7
Amazon	Bolivia	Sacta plot 1	SCT-01	0.04 ha subplots	1	-17.1	-64.8
Amazon	Bolivia	Sacta plot 6	SCT-06	mapped	1	-17.1	-64.8
Amazon	Bolivia	Tucavaca plot 1, forest	TUC-01	0.04 ha subplots	1	-18.5	-60.8
Amazon	Brazil	Alta Floresta plot 1	ALF-01	0.04 ha subplots	1	-9.6	-55.9
Amazon	Brazil	Alta Floresta plot 2	ALF-02	mapped	1	-9.6	-55.9
Amazon	Brazil	BDFFP, 2303 Dimona 5-6	BDF-01	0.04 ha subplots	2	-2.3	-60.1
Amazon	Brazil	BDFFP, 1101 Gaviao	BDF-03	0.04 ha subplots	1	-2.4	-59.9

Amazon	Brazil	BDFFP, 1102 Gaviao	BDF-04	0.04 ha subplots	1	-2.4	-59.9
Amazon	Brazil	BDFFP, 1103 Gaviao	BDF-05	0.04 ha subplots	1	-2.4	-59.9
Amazon	Brazil	BDFFP, 1201 Gaviao	BDF-06	0.04 ha subplots	3	-2.4	-59.9
Amazon	Brazil	BDFFP, 1105 Gaviao	BDF-07	0.04 ha subplots	1	-2.4	-59.9
Amazon	Brazil	BDFFP, 1109 Gaviao	BDF-08	0.04 ha subplots	1	-2.4	-59.9
Amazon	Brazil	BDFFP, 1113 Florestal	BDF-09	0.04 ha subplots	1	-2.4	-59.8
Amazon	Brazil	BDFFP, 1301 Florestal 1= plot 1301.1 and 1301.3	BDF-10	0.04 ha subplots	2	-2.4	-59.9
Amazon	Brazil	BDFFP, 1301 Florestal 2= plots 1301.4,5,6	BDF-11	0.04 ha subplots	3	-2.4	-59.8
Amazon	Brazil	BDFFP, 1301 Florestal 3=plots 1301.7,8	BDF-12	0.04 ha subplots	2	-2.4	-59.9
Amazon	Brazil	BDFFP, 3402 Cabo Frio	BDF-13	0.04 ha subplots	9	-2.4	-59.9
Amazon	Brazil	BDFFP, 3304 Porto Alegre	BDF-14	0.04 ha subplots	1	-2.4	-60.0
Amazon	Brazil	Bionte 1	BNT-01	mapped	1	-2.6	-60.2
Amazon	Brazil	Bionte 02	BNT-02	mapped	1	-2.6	-60.2
Amazon	Brazil	Bionte 4	BNT-04	mapped	1	-2.6	-60.2
Amazon	Brazil	TORRE Caxiuana	CAX-06	0.04 ha subplots	1	-1.7	-51.5
Amazon	Brazil	Caxiuana Terra Preta	CAX-08	0.04 ha subplots	1	-1.8	-51.5
Amazon	Brazil	Jacaranda, norte-sul (north-south), plots 1-5	JAC-01	0.04 ha subplots	5	-2.6	-60.2
Amazon	Brazil	Jacaranda, leste-oeste (east-west), plots 6-10	JAC-02	0.04 ha subplots	5	-2.6	-60.2
Amazon	Brazil	RESEX Chico Mendes: Seringal Porongaba 1	POR-01	0.04 ha subplots	1	-10.8	-68.8
Amazon	Brazil	RESEX Chico Mendes: Seringal Porongaba 2	POR-02	0.04 ha subplots	1	-10.8	-68.8
Amazon	Brazil	Alto Jurua	RST-01	0.04 ha subplots	1	-9.0	-72.3
Amazon	Colombia	Amacayacu: Agua Pudre E	AGP-01	0.04 ha subplots	1	-3.7	-70.3
Amazon	Colombia	Amacayacu: Agua Pudre U	AGP-02	0.04 ha subplots	1	-3.7	-70.3
Amazon	Colombia	Reserva Kalashe 1	KAL-01	0.04 ha subplots	1	11.2	-74.1
Amazon	Colombia	Amacayacu: Lorena E	LOR-01	0.04 ha subplots	1	-3.1	-70.0
Amazon	Colombia	Amacayacu: Lorena U subplot 1-13	LOR-02	0.04 ha subplots	0.52	-3.1	-70.0
Amazon	Ecuador	Jatun Sacha 2	JAS-02	0.04 ha subplots	1	-1.1	-77.6
Amazon	Ecuador	Jatun Sacha 3	JAS-03	0.04 ha subplots	1	-1.1	-77.6
Amazon	Ecuador	Jatun Sacha 4 Full plot	JAS-04	0.04 ha subplots	0.96	-1.1	-77.6
Amazon	Ecuador	Jatun Sacha 5, till 2002	JAS-05	0.04 ha subplots	1	-1.1	-77.6
Amazon	Ecuador	Tiputini 1	TIP-01	0.04 ha subplots	1	-0.7	-76.4
Amazon	Ecuador	Tiputini 2	TIP-02	0.04 ha subplots	0.8	-0.6	-76.1
Amazon	Ecuador	Tiputini 3	TIP-03	mapped	1	-0.6	-76.2
Amazon	French Guiana	Nouragues Grand Plateau 11L	NOU-02	mapped	1	4.1	-52.7
Amazon	French Guiana	Nouragues Grand Plateau 12L	NOU-03	mapped	1	4.1	-52.7
Amazon	French Guiana	Nouragues Grand Plateau 17L	NOU-08	mapped	1	4.1	-52.7

Amazon	French Guiana	Nouragues Grand Plateau 19L	NOU-10	mapped	1	4.1	-52.7
Amazon	French Guiana	Nouragues Petit Plateau 20H	NOU-11	mapped	1	4.1	-52.7
Amazon	Guyana	Forest reserve Mabura hill 01	FMH-01	mapped	1	5.2	-58.7
Amazon	Guyana	Forest reserve Mabura hill 01	FMH-03	mapped	1	5.2	-58.7
Amazon	Guyana	Pibiri 05	PIB-05	mapped	1	5.0	-58.6
Amazon	Guyana	Pibiri 06	PIB-06	mapped	1	5.0	-58.6
Amazon	Guyana	Pibiri 12	PIB-12	mapped	1	5.0	-58.6
Amazon	Peru	Altos de Maizal	ALM-01	mapped	2	-11.8	-71.5
Amazon	Peru	Allpahuayo A	ALP-01	0.04 ha subplots	1	-3.9	-73.4
Amazon	Peru	Allpahuayo B	ALP-02	0.04 ha subplots	1	-4.0	-73.4
Amazon	Peru	Allpahuayo C	ALP-30	mapped	1	-4.0	-73.4
Amazon	Peru	Allpahuayo D	ALP-40	mapped	1	-3.9	-73.4
Amazon	Peru	Cuzco Amazonico, CUZAM1E	CUZ-01	0.04 ha subplots	1	-12.5	-69.1
Amazon	Peru	Cuzco Amazonico, CUZAM1U	CUZ-02	0.04 ha subplots	1	-12.5	-69.1
Amazon	Peru	Cuzco Amazonico, CUZAM2E	CUZ-03	0.04 ha subplots	1	-12.5	-69.1
Amazon	Peru	Cuzco Amazonico, CUZAM2U	CUZ-04	0.04 ha subplots	1	-12.5	-69.1
Amazon	Peru	Jenaro Herrera A Terraza Alta	JEN-11	mapped	1	-4.9	-73.6
Amazon	Peru	Jenaro Herrera B Varillal	JEN-12	0.04 ha subplots	1	-4.9	-73.6
Amazon	Peru	Jacaratia Los Amigos	LAS-02	mapped	1	-12.6	-70.1
Amazon	Peru	Manu, alluvial Cocha Cashu Trail 3, M1	MNU-01	mapped	2.25	-11.9	-71.4
Amazon	Peru	Manu, terra firme terrace, M3	MNU-03	mapped	2	-11.9	-71.4
Amazon	Peru	Manu, terra firme ravine, M4	MNU-04	mapped	1	-11.9	-71.4
Amazon	Peru	Manu, alluvial Cocha Cashu Trail 12	MNU-05	mapped	2	-11.9	-71.4
Amazon	Peru	Manu, alluvial Cocha Cashu Trail 2 & 31	MNU-06	mapped	2.25	-11.9	-71.4
Amazon	Peru	Cocha Salvador Manu, mature floodplain	MNU-08	mapped	2	-12.0	-71.2
Amazon	Peru	Sucusari A	SUC-01	0.04 ha subplots	1	-3.3	-72.9
Amazon	Peru	Sucusari B	SUC-02	0.04 ha subplots	1	-3.2	-72.9
Amazon	Peru	Sucusari C	SUC-03	0.04 ha subplots	1	-3.2	-72.9
Amazon	Peru	Sucusari D	SUC-04	mapped	1	-3.3	-72.9
Amazon	Peru	Sucusari E	SUC-05	mapped	1	-3.3	-72.9
Amazon	Peru	Tambopata plot zero	TAM-01	0.04 ha subplots	1	-12.8	-69.3
Amazon	Peru	Tambopata plot one	TAM-02	0.04 ha subplots	1	-12.8	-69.3
Amazon	Peru	Tambopata plot three	TAM-05	0.04 ha subplots	1	-12.8	-69.3
Amazon	Peru	Tambopata plot six	TAM-07	0.04 ha subplots	1	-12.8	-69.3
Amazon	Peru	Tambopata plot seven	TAM-08	0.04 ha subplots	1	-12.8	-69.3
Amazon	Venezuela	El Dorado, KM93, plotGl, ED1	ELD-01	mapped	0.25	6.1	-61.4

Amazon	Venezuela	El Dorado, KM93, plotG2, ED1	ELD-02	mapped	0.25	6.1	-61.4
Africa	Cameroon	Bissombo 1	BIS-01	mapped	1	3.3	12.5
Africa	Cameroon	Bissombo 2	BIS-02	mapped	1	3.3	12.5
Africa	Cameroon	Bissombo 3	BIS-03	mapped	1	3.3	12.5
Africa	Cameroon	Bissombo 4	BIS-04	mapped	1	3.3	12.5
Africa	Cameroon	Bissombo 5	BIS-05	mapped	1	3.3	12.5
Africa	Cameroon	Bissombo 6	BIS-06	mapped	1	3.3	12.5
Africa	Cameroon	Campo Ma'an 1	CAM-01	0.04 ha subplots	1	2.4	9.9
Africa	Cameroon	Campo Ma'an 2	CAM-02	0.04 ha subplots	1	2.3	9.9
Africa	Cameroon	Campo Ma'an 3	CAM-03	0.04 ha subplots	1	2.4	9.9
Africa	Cameroon	Dja Somolomo 1	DJK-01	mapped	1	3.3	12.7
Africa	Cameroon	Dja Somolomo 2	DJK-02	mapped	1	3.3	12.7
Africa	Cameroon	Dja Somolomo 3	DJK-03	mapped	1	3.4	12.7
Africa	Cameroon	Dja Somolomo 4	DJK-04	mapped	1	3.4	12.7
Africa	Cameroon	Dja Somolomo 5	DJK-05	mapped	1	3.3	12.8
Africa	Cameroon	Dja Somolomo 6	DJK-06	mapped	1	3.3	12.8
Africa	Cameroon	Edjagham, plot 4	EJA-04	0.04 ha subplots	1	5.7	9.0
Africa	Cameroon	Edjagham, plot 5	EJA-05	0.04 ha subplots	1	5.7	9.0
Africa	Cameroon	Nguti plot 1	NGI-01	mapped	1	5.3	9.5
Africa	Cameroon	Nguti plot 2	NGI-02	mapped	1	5.3	9.5
Africa	Cameroon	Nguti plot 3	NGI-03	mapped	1	5.4	9.6
Africa	Gabon	Lope Reitsma	LOP-01	mapped	1	-0.2	11.4
Africa	Gabon	Monts de Cristal, plot 2	MDC-02	mapped	1	0.6	10.4
Africa	Gabon	Monts de Cristal, plot 3	MDC-03	mapped	1	0.6	10.4
Africa	Gabon	Monts de Cristal, plot 4	MDC-04	mapped	1	0.5	10.3
Africa	Ghana	Asenanyo F.R. 2	ASN-02	mapped	0.6	6.6	-2.2
Africa	Ghana	Asenanyo F.R. 4	ASN-04	mapped	0.92	6.5	-2.2
Africa	Ghana	Cape Three Points Plot 9	CAP-09	mapped	1	4.9	-2.0
Africa	Ghana	Cape Three Points Plot 10	CAP-10	mapped	1	4.8	-2.0
Africa	Ghana	Dadieso 3, occasionally flooded	DAD-31	0.04 ha subplots	0.56	6.0	-3.0
Africa	Ghana	Dadieso 4, terra firme	DAD-42	mapped	0.72	6.0	-3.0
Africa	Ghana	Fure Headwaters 7	FUR-07	mapped	1	5.6	-2.4
Africa	Liberia	Cavalla 1	CVL-01	mapped	1	6.2	-8.2
Africa	Liberia	Cavalla 8	CVL-08	mapped	1	6.2	-8.2
Africa	Liberia	Cavalla 10	CVL-10	mapped	1	6.2	-8.2
Africa	Liberia	Cavalla 11	CVL-11	mapped	1	6.2	-8.2
Africa	Liberia	Grebo F.R.1	GBO-01	mapped	1	5.4	-7.6
Africa	Liberia	Grebo F.R.2	GBO-02	mapped	1	5.4	-7.6
Africa	Liberia	Grebo F.R.3	GBO-03	mapped	1	5.4	-7.6
Africa	Liberia	Grebo F.R.4	GBO-04	mapped	1	5.4	-7.6
Africa	Liberia	Grebo F.R.8	GBO-08	mapped	1	5.4	-7.6
Africa	Liberia	Grebo F.R.10	GBO-10	mapped	1	5.4	-7.6
Africa	Liberia	Grebo F.R.11	GBO-11	mapped	1	5.4	-7.6
Africa	Liberia	Grebo F.R.13	GBO-13	mapped	1	5.4	-7.6
Africa	Liberia	Grebo F.R.14	GBO-14	mapped	1	5.4	-7.6
Africa	Liberia	Grebo F.R.15	GBO-15	mapped	1	5.4	-7.6

Africa	Liberia	Grebo F.R.16	GBO-16	mapped	1	5.4	-7.6
Africa	Liberia	Grebo F.R.18	GBO-18	mapped	1	5.4	-7.6
Africa	Liberia	Grebo F.R.19	GBO-19	mapped	1	5.4	-7.6
Africa	Tanzania	VTA-ARM-1	VTA-01	0.04 ha subplots	1	-7.8	37.0

Table S2. Comparison of individual-based tree growth and mortality models with and without effects of competition for 151 plots in Amazonia and tropical Africa. Effects of neighborhood crowding were expressed as the basal area of neighbor trees per 0.04 ha subplot (BA_{neigh}). Models were compared based on the Watanabe-Akaike Information Criterion (WAIC), with the difference from the best model (ΔWAIC) shown. Best models are indicated in bold.

	Amazon		Africa	
	growth	mortality	growth	mortality
Competition index	ΔWAIC	ΔWAIC	ΔWAIC	ΔWAIC
none	5942	2310	913	**0**
BA_{neigh}	**0**	**0**	**0**	98

Table S3. Correlations (Pearson's *r*) between the plot-level strength of competition (C_{plot}), climatic water deficit (CWD), total exchange bases (TEB), plot basal area (BA), plot wood density (WD), and mean tree size in Amazonia (*n* = 102 plots) and tropical Africa (*n* = 49 plots).

	C_{plot}	CWD	TEB	Plot BA	Plot WD
**Amazonia**					
CWD	0.58				
TEB	-0.20	0.12			
Plot BA	0.84	0.54	-0.09		
Plot WD	0.53	0.12	-0.48	0.12	
Mean tree size	0.31	0.17	-0.18	0.56	0.13
**Africa**					
CWD	0.05				
TEB	0.14	0.07			
Plot BA	0.86	0.16	0.12		
Plot WD	0.04	-0.01	0.11	0.36	
Mean tree size	0.10	0.06	-0.17	0.49	0.51

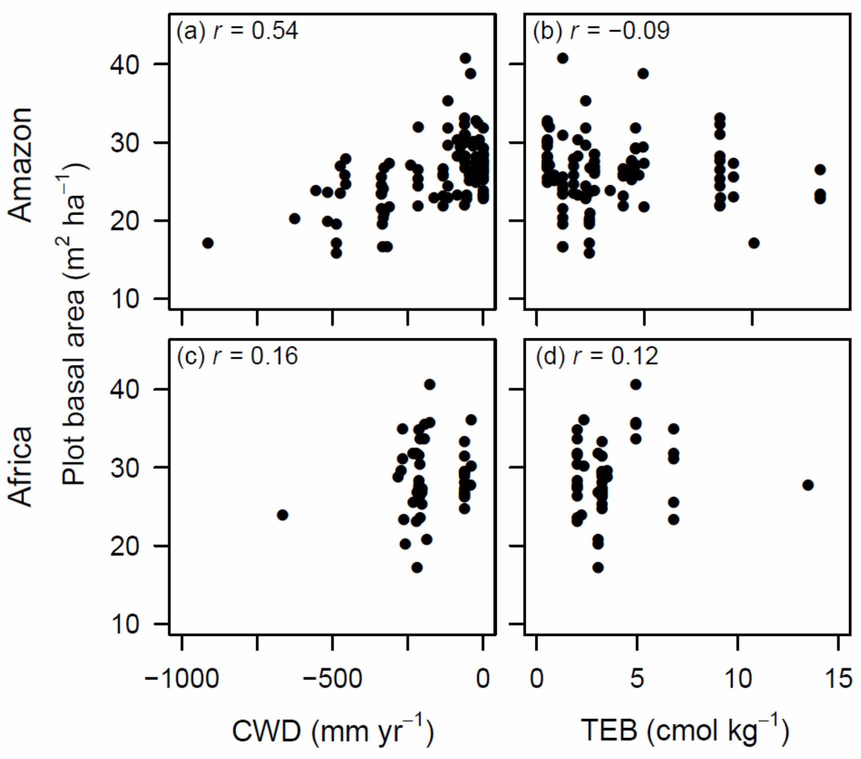

Fig. S1. Plot basal area across gradients in climatic water deficit (CWD) and soil total exchange bases (TEB) in the Amazon (*n* = 102 plots) and tropical Africa (*n* = 49 plots). Pearson's correlation coefficients (*r*) are indicated.
