# Supplementary material for: Competition influences tree growth, but not mortality, across environmental gradients in Amazonia and tropical Africa
Source: Ecology. 2020 May 5;101(7):e03052. doi: 10.1002/ecy.3052 (PMC7379300; doi:10.1002/ecy.3052)
Supplement: Supplementary file 2 — Appendix S2 [file ECY-101-e03052-s002.pdf]

Supporting Information. Rozendaal, D.M.A., O.L. Phillips, S.L. Lewis, K. Affum-Baffoe, E. Alvarez Dávila, A. Andrade, L.E.O.C. Aragão, A. Araujo-Murakami, T.R. Baker, O. Bánki, R.J.W. Brienen, J.L.C. Camargo, J.A. Comiskey, M.N. Djuikouo K., S. Fauset, T.R. Feldpausch, T.J. Killeen, W.F. Laurance, S.G.W. Laurance, T. Lovejoy, Y. Malhi, B.S. Marimon, B.-H. Marimon Junior, A.R. Marshall, D.A. Neill, P. Núñez Vargas, N.C.A. Pitman, L. Poorter, J. Reitsma, M. Silveira, B. Sonké, T. Sunderland, H. Taedoumg, H. ter Steege, J.W. Terborgh, R.K. Umetsu, G.M.F. van der Heijden, E. Vilanova, V. Vos, L.J.T. White, S. Willcock, L. Zemagho, and M.C. Vanderwel. 2020. Competition influences tree growth, but not mortality, across environmental gradients in Amazonia and tropical Africa. Ecology.

Detailed description of individual growth and mortality models

Appendix S2

Individual growth and mortality models

We modelled tree growth and mortality as a function of size, competition and wood density for each continent (Amazonia, tropical Africa), using non-linear hierarchical models. Annual basal area growth (G) and annual probability of mortality (M) for a tree were modeled as follows:

$$G = a_G \times p_G \times S_G \times C_G$$
$$M = [1 + a_M \times p_M \times S_M \times C_M]^{-1}$$

where a_G and a_M are constants, and p_G and p_M are plot-level random effects. S and C (for growth and mortality) are non-linear functions that capture effects of tree size and competition:

$$S = \mathrm{dbh}^{s_1} \times \exp(-s_2 \times \mathrm{dbh})$$
$$C = \exp(-c_1 \times \mathrm{dbh}^{c_2} \times \mathrm{BA}_{\mathrm{neigh}})$$

where dbh indicates diameter at breast height of a tree, and $\mathrm{BA}_{\mathrm{neigh}}$ its neighbor basal area. s_1, s_2, c_1 and c_2 control the shape of the functions and have separate values for growth and mortality. To aid model fitting, we re-scaled dbh and $\mathrm{BA}_{\mathrm{neigh}}$ by dividing them by numbers close to their observed means.

We accounted for species-to-species variation in growth and mortality by constructing models based on WD. To incorporate WD effects, we defined a, s_1, s_2, c_1 and c_2 as linear functions of WD. For each of these (here θ), we estimated parameters θ_{min} and θ_{max} to define their values at the minimum and maximum WD that was observed among species ($\mathrm{WD}_{\mathrm{min}}$ and $\mathrm{WD}_{\mathrm{max}}$, respectively). We used linear interpolation to estimate θ for each stem as:

$$\theta = w\theta_{\mathrm{min}} + (1-w)\theta_{\mathrm{max}}$$

where weighting coefficient w is calculated as:

$$w = \frac{\mathrm{WD}_{\mathrm{max}} - \mathrm{WD}}{\mathrm{WD}_{\mathrm{max}} - \mathrm{WD}_{\mathrm{min}}}$$

We used hierarchical Bayesian modeling to estimate the posterior distribution of the parameters for each model. Annual basal area growth (ΔBA) of individual trees was modeled by a normal distribution, with a standard deviation that increased as a linear function of dbh. Observations of tree survival (D=0) or death (D=1) over the census interval were modeled by a Bernoulli distribution whose mean was the compounded probability of mortality over a census interval of y years. Plot effects on tree growth and mortality were considered to be normally distributed with a mean of one:

$$\Delta \mathrm{BA} \sim \mathrm{N}(G, \sigma_0 + \sigma_{\mathrm{dbh}} \times \mathrm{dbh})$$
$$D \sim \mathrm{Bern}(1 - (1 - M)^y)$$
$$p_\mathrm{G} \sim \mathrm{N}(1, \sigma_\mathrm{G})$$
$$p_\mathrm{M} \sim \mathrm{N}(1, \sigma_\mathrm{M})$$

where σ_0, σ_{dbh}, σ_G, and σ_M are estimated parameters. All model parameters were assigned uninformative priors. We performed Markov chain Monte Carlo sampling with four chains, with per chain 750 000 burn-in and 500 000 sampling iterations. We thinned chains by retaining every 100th sample, and assessed convergence using the Gelman-Rubin criterion (Gelman and Rubin 1992). To assess whether competition influenced growth and mortality, we compared the model with effects of competition with a model that excluded competition by fixing either C_G or C_M at 1, based on the Watanabe-Akaike Information Criterion (WAIC; Watanabe 2013, Hooten and Hobbs 2015).

All analyses were performed in R 3.1.2 (R Core Team 2014). MCMC sampling was performed using the 'Filzbach' sampler, as implemented in the 'filzbach' package (Lyutsarev and Purves 2013). The Gelman-Rubin criterion was calculated using the 'coda' package (Plummer et al. 2006).

Comparison of indices of competition

To assess whether $\mathrm{BA}_{\mathrm{neigh}}$ accurately captured local effects of competition, we also fit models with a neighborhood crowding index (NCI; see Uriarte et al. 2004) instead of $\mathrm{BA}_{\mathrm{neigh}}$ for a subset of 86 plots for which at least 90 % of the trees were mapped. We randomly assigned coordinates to trees that were not mapped within the corresponding 0.04 ha subplot. All trees within 10 m of the edge of the plot were excluded from analysis, as their neighborhood is partially undefined. NCI is considered to be a good proxy for light availability to an individual tree (Grote et al. 2013). NCI is based on the size of and distance to, neighbor trees within a 10 m radius of the focal tree:

$$\mathrm{NCI} = \sum_{j=1, i \neq j}^{J} \frac{\mathrm{dbh}_j^2}{\mathrm{dist}_{ij}^2}$$

where dist is the distance between focal tree i and neighbor tree j for a total of J neighbor trees. For both continents, growth models that included NCI were not better supported than models with $\mathrm{BA}_{\mathrm{neigh}}$ (Table S1). A mortality model that included NCI performed better than a model with $\mathrm{BA}_{\mathrm{neigh}}$ for Africa, but for Amazonia the mortality model without competition was best supported (Table S1). Thus, $\mathrm{BA}_{\mathrm{neigh}}$ generally captured local effects of competition, but not for tree mortality in Africa. Nevertheless, also in models based on NCI, effects of competition on

mortality were negligible (results not shown). We therefore included results for models that included BA_{neigh} only, based on data from all 151 plots.

Table S1. Comparison of individual-based tree growth and mortality models that varied in competition index for 86 plots in Amazonia (*n* = 44) and tropical Africa (*n* = 42) for which trees were mapped. Models were compared based on the Watanabe-Akaike Information Criterion (WAIC), with the difference from the best model (ΔWAIC) shown. Best models are indicated in bold. NCI = neighborhood crowding index; BA_{neigh} = basal area of neighbor trees per 0.04 ha subplot.

	Amazon		Africa	
	growth	mortality	growth	mortality
Competition index	ΔWAIC	ΔWAIC	ΔWAIC	ΔWAIC
none	1566	**0**	1079	384
NCI	177	437	761	**0**
BA_{neigh}	**0**	331	**0**	295

Literature Cited

Gelman, A., and D. B. Rubin. 1992. Inference from iterative simulation using multiple sequences. Statistical Science **7**:457-511.

Grote, S., R. Condit, S. Hubbell, C. Wirth, and N. Rüger. 2013. Response of demographic rates of tropical trees to light availability: Can position-based competition indices replace information from canopy census data? Plos One **8**.

Hooten, M. B., and N. T. Hobbs. 2015. A guide to Bayesian model selection for ecologists. Ecological Monographs **85**:3-28.

Lyutsarev, V., and D. Purves. 2013. filzbach: Filzbach MCMC sampler. R package version 0.6-1. https://github.com/predictionmachines/Filzbach.

Plummer, M., N. Best, K. Cowles, and K. Vines. 2006. CODA: Convergence diagnosis and output analysis for MCMC. R News **6**:7-11.

R Core Team. 2014. R: A language and environment for statistical computing. R Foundation for Statistical Computing, Vienna, Austria.

Uriarte, M., C. D. Canham, J. Thompson, and J. K. Zimmerman. 2004. A neighborhood analysis of tree growth and survival in a hurricane-driven tropical forest. Ecological Monographs **74**:591-614.

Watanabe, S. 2013. A widely applicable bayesian information criterion. Journal of Machine Learning Research **14**:867-897.
