# Supplementary material for: Competition influences tree growth, but not mortality, across environmental gradients in Amazonia and tropical Africa
Source: Ecology. 2020 May 5;101(7):e03052. doi: 10.1002/ecy.3052 (PMC7379300; doi:10.1002/ecy.3052)
Supplement: Supplementary file 3 — Metadata S1 [file ECY-101-e03052-s004.pdf]

Rozendaal, D.M.A., O.L. Phillips, S.L. Lewis, K. Affum-Baffoe, E. Alvarez Dávila, A. Andrade, L.E.O.C. Aragão, A. Araujo-Murakami, T.R. Baker, O. Bánki, R.J.W. Brienen, J.L.C. Camargo, J.A. Comiskey, M.N. Djuikouo K., S. Fauset, T.R. Feldpausch, T.J. Killeen, W.F. Laurance, S.G.W. Laurance, T. Lovejoy, Y. Malhi, B.S. Marimon, B.-H. Marimon Junior, A.R. Marshall, D.A. Neill, P. Núñez Vargas, N.C.A. Pitman, L. Poorter, J. Reitsma, M. Silveira, B. Sonké, T. Sunderland, H. Taedoumg, H. ter Steege, J.W. Terborgh, R.K. Umetsu, G.M.F. van der Heijden, E. Vilanova, V. Vos, L.J.T. White, S. Willcock, L. Zemagho, and M.C. Vanderwel. 2020. Competition influences tree growth, but not mortality, across environmental gradients in Amazonia and tropical Africa. Ecology.

Data S1

R code for the growth and the mortality model.

Authors

Danaë M.A. Rozendaal
Plant Production Systems Group and Centre for Crop Systems Analysis, Wageningen University
P.O. Box 430, 6700 AK Wageningen, the Netherlands
danae.rozendaal@wur.nl

Mark C. Vanderwel
Deparment of Biology, University of Regina
3737 Wascana Parkway, Regina, Saskatchewan, Canada
mark.vanderwel@uregina.ca

File list

`Model_script.R`

Description

Models were fit using the 'filzbach' R package. The code for the models for Amazonia is indicated (102 plots), for Africa the same models were used. Model parameters are explained in Appendix S2.

The dataframe "data" includes treelevel data, the following columns were included in the models:

WD - tree wood density (g cm^{-3})
dbh - tree diameter at breast height in the first census (cm)
neighBA - basal area of neighbour trees in the 0.04-ha subplot (m^2 ha^{-1})
PlotCode - plot identifier
BAgrowth - annual basal area growth (cm^2 yr^{-1})
dead - 1 indicates that the tree is dead, 0 indicates that the tree is alive
IntervalLength - length of the census interval (yr)

`Model_script.R` - File including the model code.
